# Lung Ultrasound to Diagnose Pneumonia in Neonates with Fungal Infection

**DOI:** 10.3390/diagnostics12081776

**Published:** 2022-07-22

**Authors:** Jing Liu, Hai-Ran Ma, Wei Fu

**Affiliations:** 1 Department of Neonatology and NICU, Beijing Chao-Yang Hospital, Capital Medical University, Beijing 100043, China; 2 Department of Neonatology and NICU, Beijing Chao-Yang District Maternal and Child Healthcare Hospital, Beijing 100021, China; fuweibj81@126.com; 3 Department of Neonatology and NICU, Huizhou Municipal Central Hospital, Huizhou 516000, China; hzzxmhr@163.com

**Keywords:** lung ultrasound, lung disease, pneumonia, fungus, newborn infant, chest X-ray

## Abstract

With the improvement in survival rates of low-birthweight and very premature infants, neonatal fungal infection, especially fungal pneumonia, is becoming more and more common, but the diagnosis is always challenging. Recently, lung ultrasound (LUS) has been used to diagnose pneumonia in newborn infants, but not fungal pneumonia. This paper summarizes the ultrasonographic features of seven cases of neonatal fungal pneumonia, such as lung consolidation with air bronchograms, shred signs, lung pulse, pleural line abnormalities, and different kinds of B-lines. It was confirmed that LUS plays an important role in the diagnosis of fungal pneumonia in newborn infants.

## 1. Introduction 

Pneumonia is one of the most common lung diseases in newborn infants and one of the major causes of neonatal death; bacteria and viruses are the most common pathogens of neonatal infectious pneumonia [1]. With the improvement in survival rates of low-birthweight and very premature infants, neonatal fungal infection (FI), especially fungal pneumonia (FP), is becoming more and more common [2,3,4,5]. It was reported that the overall incidence of FI is 2.4/1000 neonatal unit admissions and 18.8/1000 among infants weighing < 1000 g [2]. The incidence of FI was reported as 7.3% in Korea [3], 0.33 per 1000 live births of hospitalized neonates in Japan [4], and 0.74 per 1000 preterm infants discharged from NICU in China [5]. The fatality rate of neonatal FI infection is also high; it was reported that the total fatality rate is 21% and the incidence can be as high as 40% in infants with a birth weight of <1500 g [2,3]. Timely and accurate diagnosis is important to enable efficient treatment and improve the prognosis of patients with neonatal pneumonia. Recently, lung ultrasound (LUS) has been successfully used to diagnose several kinds of neonatal lung diseases, such as meconium aspiration syndrome (MAS) [6,7], respiratory distress syndrome (RDS) [8,9], transient tachypnea of newborn (TTN) [10,11], pneumothorax [12,13], pulmonary hemorrhage [14], bronchopulmonary dysplasia (BPD) [15], bronchiolitis [16], and congenital lung malformation [17,18]. LUS has especially been widely used in the diagnosis of neonatal pneumonia, including bacterial or viral pneumonia, but not including fungal pneumonia [19,20,21,22,23]. Since March 2017, LUS has completely replaced chest X-ray (CXR) for routine diagnosis and differential diagnosis of neonatal lung disease in our neonatal intensive care unit (NICU) [24], and during this period, FP was diagnosed seven times. This paper summarizes the ultrasound imaging characteristics of FP to help clinicians use ultrasound technology to make early and accurate diagnoses of neonatal FP.

## 2. Patients and Methods 

### 2.1. Patients 

The study protocol was approved by the ethics committee of Beijing Chaoyang District Maternal and Child Healthcare Hospital (No.2011-LC-Ped-01). Written informed consent was obtained from the participants’ parents. LUS was performed according to the relevant guidelines and regulations [25,26].

Seven newborn infants diagnosed with FP by the clinical team according to the criteria described below were included in this study. There were 5 male and 2 female infants; 4 of them were delivered by cesarean section and 3 were delivered vaginally. All of the patients were premature infants; the gestational age was from 27^+1^ to 35^+4^ weeks, and the birth weight was from 880 g to 4200 g. Twenty premature infants with no lung disease admitted to the same NICU during the same period were included as controls. There were 12 male and 8 female infants in the control group. The gestational age was from 28 weeks to 35^+1^ weeks with a birth weight of 900 to 3900 g. The major reasons for admission were low birth weight, poor feeding, jaundice, or sepsis, but not lung conditions. All LUS examinations were performed by the examiners who were blinded to the clinical diagnosis. 

### 2.2. Lung Ultrasound Examination

#### 2.2.1. Equipment

The GE Voluson S10 (GE Healthcare, Chicago, IL, USA) ultrasound system with a linear array probe (frequency > 10 MHz) was used for LUS examinations. All of the examinations were strictly performed in accordance with published guidelines and specifications [25,26].

#### 2.2.2. LUS Examination Method

While in a quiet state, infants were positioned in the supine, lateral, or prone position. Each lung was divided into 6 regions, which were the anterior, lateral and posterior regions by the anterior axillary line, posterior axillary line and nipple connection line. Each region of both lungs was scanned carefully with the probe perpendicular to and parallel to the ribs [25,26].

#### 2.2.3. Observation Indexes

The observation indexes included pleural lines, A-lines, B-lines, lung consolidation with air bronchograms, shred signs, and pleural effusion. These were defined as follows [11]: (1) Pleural line: the regular echogenic line under the superficial layers of the thorax moving continuously during respiration, while abnormal pleural lines refer to pleural line disappearance, thickening, irregularity, or a coarse and indistinct appearance. (2) Lung sliding: in real-time ultrasound, we find that the pleural line moves in a to-and-fro pattern, synchronized with respiratory movement, which is called lung sliding. (3) A-line: a series of echogenic, horizontal, parallel lines equidistant from one another below the pleural line. (4) B-lines, also known as ultrasound lung comets: hyperechoic narrow-based artifacts spreading in a similar way to laser rays from the pleural line to the edge of the screen. (5) Lung consolidation: areas of hepatization with the presence of air bronchograms and/or fluid bronchograms. (6) Air bronchograms: the hyperechoic reflection within the region of consolidation. (7) Shred signs: the hyper echoic reflection located at the edge of the region of consolidation. (8) Pleural effusion: anechoic-dependent collections limited by the diaphragm and the pleura. (9) Lung pulse: when the lung consolidation is large enough and near the edges of the heart, the consolidated lung may appear to be pulsating in synchrony with the heartbeat under real-time ultrasound. This kind of movement is called the lung pulse.

#### 2.2.4. Statistical Analysis 

Data analyses were performed using SPSS version 24.0 (IBM Inc; Armonk, NY, USA) for Windows. Positive ultrasound findings were compared between the two groups using Fisher’s exact test. We accepted *p* < 0.05 as indicating statistical significance. 

## 3. Results

### 3.1. Clinical Manifestations of Fungal Pneumonia

Fungal pneumonia was diagnosed by the clinical staff according to the following criteria [2,3,4,5]: (1) Presence of fever, dyspnea, irregular breathing, or other respiratory symptoms. (2) Fine moist rales on auscultation. (3) Significantly increased or decreased white blood cell count and/or high C-reactive protein (CRP) level as well as markedly decreased platelet count. (4) Blood culture and/or deep sputum culture were/was positive for fungi, as well as a positive 1, 3-β-d-glucan test (G test). Patients were excluded if there was no definite evidence of fungal infection, or a combination of bacterial or viral infection simultaneously, or if they had serious complications such as pulmonary hemorrhage, etc., that might disturb the LUS findings.

All seven cases met the above criteria. The onset of illness occurred 10–21 days after birth, except for one infant whose onset occurred on the second day after birth. Every patient suffered from severe dyspnea, four had fever; CRP significantly increased and platelet counts significantly decreased in all seven infants. The 1, 3-β-d-glucan test (G test) was positive in all seven patients. The results of blood and/or deep sputum culture showed *Candida albicans* in 5 patients, *Candida parapsilosis* in 1 patient, and aspergillus in 1 patient. All of the patients received antifungal therapy; two had fluconazole and the remaining five had voriconazole or caspofungin. Five patients were treated with invasive ventilation and two were treated with non-invasive ventilation. Six patients recovered and were discharged, while one of them died due to pneumothorax, subcutaneous emphysema, and systemic multiple organ failure, which resulted in a fatality rate of 14.28%.

### 3.2. Ultrasound Manifestation of Neonatal Normal Lung

**Figure 1:**
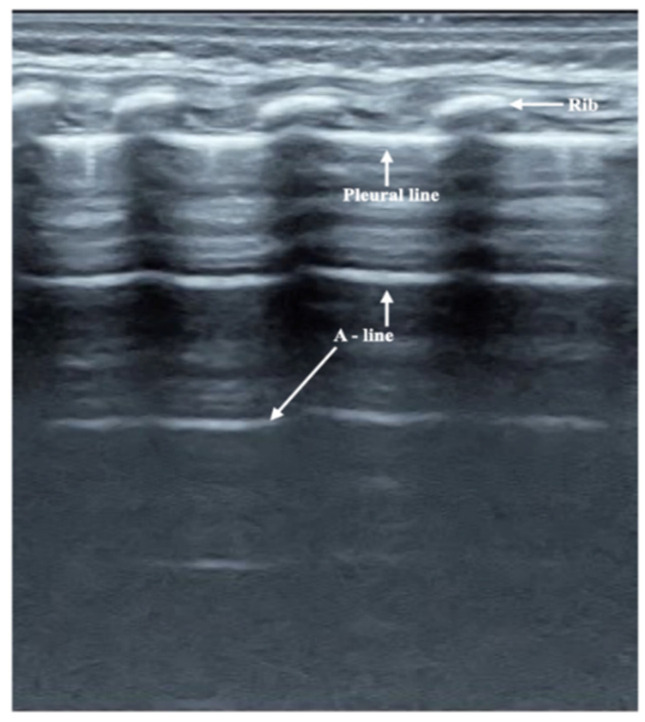
Normal Lung Ultrasound Manifestation. On B-mode ultrasound, the pleural line and A-line were parallel to each other, which formed a kind of bamboo-like ultrasound image, i.e., the bamboo sign.

On B-mode ultrasound (Figure 1), the pleural line and A-line show smooth, regular and hyperechoic lines arranged in parallel and equidistant from each other. The A-line echoes gradually diminish until they disappear. Together, they form a kind of bamboo-like ultrasound image called a bamboo sign. There were a few (within 72 h after birth) or no B-lines (72 h after birth). There was no lung consolidation or pleural effusion in any of the controls. However, under real-time ultrasound, a clear pleural line moving with the respiratory movement, i.e., lung sliding, could be seen in all of the infants (Video S1).

### 3.3. LUS Findings of FP

The major ultrasound findings of the seven FP cases were as following: (1) All of the seven patients presented with significant lung consolidation with air bronchograms as well as irregular or jagged boundaries. Generally, the consolidation could be found in bilateral lung fields. Three of them developed into significant atelectasis. (2) Shred signs were visible at the edges of the consolidated areas in four severe patients. (3) Pleural line abnormalities, including disappearance, irregularity, disruption, and coarse appearance, were found in all seven infants. (4) Different kinds of B-lines in the non-consolidated area meant that different degrees of lung edema co-existed. (5) Lung pulse was found in two patients (Video S2). (6) Pleural effusion was found in two patients. The detailed lung ultrasound findings of FP are shown in Table 1.

### 3.4. Typical Case Presentations

To our knowledge, the use of ultrasound to diagnose neonatal FP has not been described before. In order to help clinicians better understand the ultrasonic manifestations of FP, two typical cases are introduced here.

**Figure 2:**
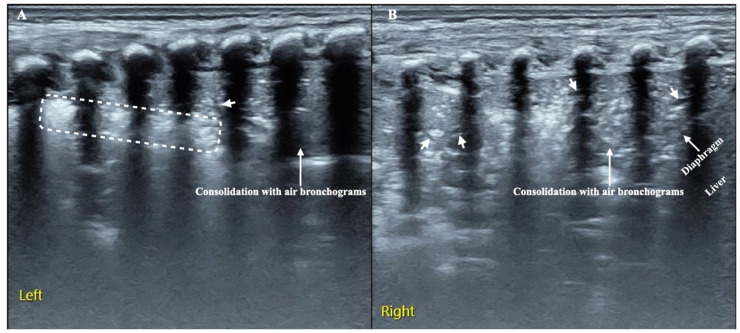
LUS manifestations of neonatal fungal pneumonia. It can be seen from the picture that both lungs have massive consolidation with air bronchograms, which resulted in severe atelectasis. Shred signs can be seen at the edge of the consolidation area. In addition, there are some minor fluid bronchograms can be found in bilateral lung fields (arrows). (**A**): left lung; (**B**): right lung. (Arrows: lung consolidation with air bronchograms. Dotted box: shred signs).

**Case 1.** This is a male premature infant delivered vaginally at gestational age 27^+1^ weeks with a birth weight of 890 g and no birth asphyxia, who was hospitalized at the NICU due to severe respiratory distress 30 min after birth. His mother suffered from fungal vaginitis, and three consecutive cultures of vaginal secretions showed *Candida albicans* growth. On admission, physical examination showed significant respiratory difficulty, which presented as a respiratory frequency of 85 breaths/min accompanied by grunting, flaring, and retracting. On the second day after admission, both blood culture and deep sputum culture showed *Candida albicans* growth, and the G test result was positive. The LUS showed a large area of lung consolidation with air bronchograms in both lungs, with the lung consolidation involving the entire field of his right lung. In addition, there were obvious shred signs at the edge of the consolidation area of the left lung (Figure 2).

**Figure 3:**
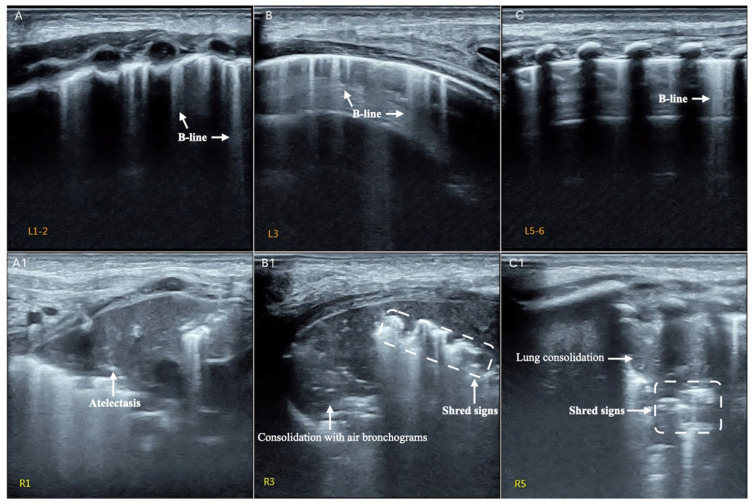
LUS manifestations of neonatal fungal pneumonia. It can be seen from the picture that the left lung mainly shows a few B-lines, suggesting the presence of mild lung edema. In the right lung, there was obvious consolidation with air bronchograms and shred signs in the subaxillary and posterior areas. Atelectasis was seen in the upper lung field of the right anterior chest. (**A**): the left anterior lung field, (**B**): the left subaxillary lung field and (**C**): the left posterior upper lung field. (**A1**): the right anterior upper field, (**B1**): right subaxillary upper lung field and (**C1**): right posterior upper lung field.

**Case 2.** This is a male premature infant delivered vaginally at gestational age 35^+4^ weeks with a birth weight of 2900 g with no birth asphyxia, who was hospitalized at the NICU due to RDS. Fever was present, and white blood cell count (25 × 10^9^/L) and neutrophil proportion (>90%) were significantly increased. The platelet count was <10 × 10^9^/L and CRP was >100 mg/L within 24 h after birth. The infant had several complications, such as diffuse intravascular coagulation, capillary leakage syndrome, gas leakage syndrome and persistent pulmonary hypertension. Therefore, the infant was treated with invasive ventilation and broad-spectrum antibiotics for more than 2 weeks. On day 20 after birth, the infant developed severe dyspnea and fever again. LUS showed several B-lines as the main manifestation in the left lung, but significant lung consolidation and atelectasis were found in his right anterior upper field, right subaxillary upper lung field and right posterior upper lung field. Subsequently, both the blood cultures and deep sputum cultures confirmed this baby had *Candida albicans* infection (Figure 3).

## 4. Discussion

Generally, neonatal pneumonia is divided into three subtypes, depending on the time of onset, which are congenital (or intrauterine) pneumonia (infection established during fetal life), early-onset pneumonia (develops within the first week of life) and late-onset pneumonia (develops after the first week of life, including ventilator-associated pneumonia). According to this classification method, most of the neonatal FP may belong to late-onset pneumonia [1]. Preterm birth, low birth weight and long-term use of broad-spectrum antibiotics are the main causes of neonatal fungal pneumonia [3,4,5].

The diagnosis of neonatal pneumonia is always a challenge. Compared to older children and adults, neonates show fewer localized signs of pneumonia. For a long time, in addition to hematological indicators (such as peripheral blood parameters, blood culture, etc.) [27,28], CXR examination has been the most important method to diagnose neonatal pneumonia [1], which mainly presents as increased and thickened lung texture, speckled small or larger patchy shadow, and may be accompanied by atelectasis or local emphysema in different lung fields. However, X-ray manifestations of neonatal pneumonia are not specific, with poor accuracy and reliability, and will inevitably cause radiation damage to infants. Recently, LUS has been successfully used for the diagnosis and differential diagnosis of neonatal lung disease, including infectious pneumonia [19,20,21,22,23], and it was confirmed that LUS has greater accuracy and reliability in the diagnosis of neonatal lung disease and neonatal pneumonia than CXR [29,30]. The results from a meta-analysis showed that the sensitivity was 96% and the specificity was 98% by using LUS to diagnose neonatal pneumonia, which was much higher than that of CXR, where the sensitivity and specificity was only 74% and 92%, respectively [30]. Since LUS contributes to the early diagnosis of neonatal FP, it can help clinicians make early treatment decisions and therefore improve the patient prognosis. In this study, only one infant died due to complicated severe multiple organ injury, giving a fatality rate of 14.28%. This is significantly lower than previously reported fatality rates of 21~40% [2,3]. Therefore, LUS technology is worthy of extensive development and application in NICU. Of course, the correct selection of antifungal agents is also one of the important measures for improving neonatal prognosis. Although fluconazole is most commonly used as a first-line agent for the treatment of neonatal fungal infections [4,5,31,32], resistance has increased in recent years. In this study, two patients were treated with fluconazole; however, one died, while the remaining five patients were fully cured after treatment with voriconazole or caspofungin.

The results of this study showed that LUS is very helpful in the diagnosis of neonatal fungal pneumonia. Lung consolidation with air bronchograms as well as irregular or jagged boundaries are the most important LUS characteristics. Lung consolidation may involve all lung fields of both lungs (Figure 1) or only involve part of the intercostal space (Figure 2). Other common findings are abnormal pleural lines or pulmonary edema and shred signs, and a small minority of infants (28.6% in this study) may have a pleural effusion. Lung pulse is a rare ultrasound manifestation, but its occurrence suggests a large area of lung consolidation and severe disease. If the lung lesion develops into a large area with regular boundary consolidation, atelectasis is indicated [33]. When a premature infant, especially an extremely low birth weight or extremely premature infant, receives broad spectrum antibiotics in hospital but fever and breathing difficulty persist, one should think of the possibility of fungal pneumonia, especially when the above typical LUS findings are present. Although fluid bronchogram is seen in pneumonia, it is most common in pulmonary hemorrhage patients, according to our experience. In addition, pneumonia caused by various pathogens may be difficult to identify by ultrasound imaging [19,20]; therefore, timely blood culture, deep sputum culture or G test should be taken to confirm the diagnosis. There were 2 patients (28.6%) with signs of this in our study. For infants with severe lung consolidation and atelectasis, bronchoalveolar lavage may be necessary for recovery [34,35].

## 5. Limitation

Some limitations remained in this study. The major limitation lies in the small number of cases in this paper, which may cause deviations in the results. Therefore, the ultrasound imaging characteristics of fungal pneumonia remain to be summarized in a large sample of cases. Second, LUS cannot accurately determine the etiology of pneumonia; the exact etiological diagnosis still depends on serological examination. Third, only those appropriately trained physicians are likely to perform lung ultrasound technology [25,26,36].

## 6. Conclusions

In conclusion, this paper describes the ultrasound imaging features of several cases of neonatal FP confirmed by etiological examination. The most common and important LUS feature is air bronchograms, which show areas of lung consolidation, while atelectasis and lung pulse could be seen in severe patients. Mastering lung ultrasound technology will be helpful for the early diagnosis and differential diagnosis of neonatal FP, so as to improve the prognosis of newborn infants.

## Figures and Tables

**Table 1:** Ultrasound manifestation in Neonatal fungal pneumonia (n,%).

Ultrasound Manifestation	Pneumonia (n = 7)	Controls (n = 20)	*p*-Value
Lung consolidation	7 (100)	0 (0)	<0.001
Pleural line abnormalities	7 (100)	0 (0)	<0.001
Pleural effusion	2 (28.6)	0 (0)	<0.001
Lung pulse	2 (28.6)	0 (0)	<0.001
B-lines	7 (100)	3 (15)	<0.001

## Data Availability

Not applicable.

## Supplementary Materials

The following supporting information can be downloaded at: https://www.mdpi.com/article/10.3390/diagnostics12081776/s1. Video S1: Lung sliding. In real-time ultrasound, we can see the pleural line moving with the respiratory movement, i.e., lung sliding. Video S2. Lung Pulse. Lung consolidation with air bronchograms are seen, with the thoracic aorta lying below the area of consolidation. In real-time ultrasound, we can see the large consolidated area of lung pulsate with the movement of the heartbeat, which is known as lung pulse.

## Abbreviations

LUS	lung ultrasound
NICU	neonatal intensive care unit
CXR	chest X-ray
NFI	neonatal fungal infection
FP	fungal pneumonia
MAS	meconium aspiration syndrome
RDS	respiratory distress syndrome
TTN	transient tachypnea of newborn
BPD	bronchopulmonary dysplasia
CRP	c-reactive protein
G test	1, 3-β-d-glucan test

## References

[1] Hooven T.A., Polin R.A. (2017). Pneumonia. Semin. Fetal Neonatal Med..

[2] Oeser C., Vergnano S., Naidoo R., Anthony M., Chang J., Chow P., Clarke P., Embleton N., Kennea N., Pattnayak S. (2014). Neonatal invasive fungal infection in England 2004–2010. Clin. Microbiol. Infect..

[3] Jung Y.J. (2020). Risk factors for fungal infection in extremely low birthweight infants registered in the Korean neonatal network from 2013 to 2015: Male sex and hypotension. Pediatr. Int..

[4] Ishiwada N., Kitajima H., Morioka I., Takeuchi N., Endo M., Watanabe A., Kamei K. (2018). Nationwide survey of neonatal invasive fungal infection in Japan. Med. Mycol..

[5] Xia H., Wu H., Xia S., Zhu X., Chen C., Qiu G., Zhou W., Shi Y., Mia L., Sun J. (2014). Invasive candidiasis in preterm neonates in China: A retrospective study from 11 NICUs during 2009–2011. Pediatr. Infect. Dis. J..

[6] Piastra M., Yousef N., Brat R., Manzoni P., Mokhtari M., De Luca D. (2014). Lung ultrasound findings in meconium aspiration syndrome. Early Hum. Dev..

[7] Liu J., Cao H.-Y., Fu W. (2016). Lung ultrasonography to diagnose meconium aspiration syndrome of the newborn. J. Int. Med. Res..

[8] Liu J., Cao H.Y., Wang H.-W., Kong X.Y. (2015). The Role of Lung Ultrasound in Diagnosis of Respiratory Distress Syndrome in Newborn Infants. Iran. J. Pediatr..

[9] Ma H., Yan W., Liu J. (2020). Diagnostic value of lung ultrasound for neonatal respiratory distress syndrome: A meta-analysis and systematic review. Med. Ultrason..

[10] Liu J., Chen X.-X., Li X.-W., Wang Y., Chen S.-W., Fu W. (2016). Lung Ultrasonography to Diagnose Transient Tachypnea of the Newborn. Chest.

[11] He L., Sun Y., Sheng W., Yao Q. (2021). Diagnostic performance of lung ultrasound for transient tachypnea of the newborn: A meta-analysis. PLoS ONE.

[12] Liu J., Chi J.-H., Ren X.-L., Li J., Chen Y.-J., Lu Z.-L., Liu Y., Fu W., Xia R.-M. (2017). Lung ultrasonography to diagnose pneumothorax of the newborn. Am. J. Emerg. Med..

[13] Szymońska I., Wentrys L., Jagła M., Olszewska M., Wasilewska W., Smykla B., Kwinta P. (2019). Lung ultrasound reduces the number of chest X-rays in newborns with pneumothorax. Dev. Period. Med..

[14] Ren X.-L., Fu W., Liu J., Liu Y., Xia R.-M. (2016). Lung ultrasonography to diagnose pulmonary hemorrhage of the newborn. J. Matern. Fetal Neonatal Med..

[15] Liu J., Chi J.-H., Fu W., Zhang L., Qiu R.-X. (2021). Lung Ultrasonography to Diagnose Bronchopulmonary Dysplasia of Premature Infants. Iran. J. Pediatr..

[16] Di Mauro A., Ammirabile A., Quercia M., Panza R., Capozza M., Manzionna M.M., Laforgia N. (2019). Acute Bronchiolitis: Is There a Role for Lung Ultrasound?. Diagnostics.

[17] Quercia M., Panza R., Calderoni G., Di Mauro A., Laforgia N. (2019). Lung Ultrasound: A New Tool in the Management of Congenital Lung Malformation. Am. J. Perinatol..

[18] Merli L., Nanni L., Curatola A., Pellegrino M., De Santis M., Silvaroli S., Paradiso F.V., Buonsenso D. (2021). Congenital lung malformations: A novel application for lung ultrasound?. J. Ultrasound.

[19] Liu J., Liu F., Liu Y., Wang H.-W., Feng Z.-C. (2014). Lung Ultrasonography for the Diagnosis of Severe Neonatal Pneumonia. Chest.

[20] Öktem A., Zenciroğlu A., Üner Ç., Aydoğan S., Dilli D., Okumuş N. (2021). Efficiency of Lung Ultrasonography in the Diagnosis and Follow-up of Viral Pneumonia in Newborn. Am. J. Perinatol..

[21] Gregorio-Hernández R., Escobar-Izquierdo A.B., Cobas-Pazos J., Martínez-Gimeno A. (2020). Point-of-care lung ultrasound in three neonates with COVID-19. Eur. J. Pediatr..

[22] Stoicescu E.R., Ciuca I.M., Iacob R., Iacob E.R., Marc M.S., Birsasteanu F., Manolescu D.L., Iacob D. (2021). Is Lung Ultrasound Helpful in COVID-19 Neonates?—A Systematic Review. Diagnostics.

[23] Tusor N., De Cunto A., Basma Y., Klein J.L., Meau-Petit V. (2021). Ventilator-associated pneumonia in neonates: The role of point of care lung ultrasound. Eur. J. Pediatr..

[24] Gao Y.-Q., Qiu R.-X., Liu J., Zhang L., Ren X.-L., Qin S.-J. (2020). Lung ultrasound completely replaced chest X-ray for diagnosing neonatal lung diseases: A 3-year clinical practice report from a neonatal intensive care unit in China. J. Matern. Fetal Neonatal Med..

[25] Liu J., Copetti R., Sorantin E., Lovrenski J., Rodriguez-Fanjul J., Kurepa D., Feng X., Cattaross L., Zhang H., Yeh T.F. (2019). Protocol and Guidelines for Point-of-Care Lung Ultrasound in Diagnosing Neonatal Pulmonary Diseases Based on International Expert Consensus. J. Vis. Exp..

[26] Liu J., Guo G., Kurepa D., Volpicelli G., Sorantin E., Lovrenski J., Alonso-Ojembarrena A., Hsieh K.-S., Lodha A., Yeh T.F. (2021). Specification and guideline for technical aspects and scanning parameter settings of neonatal lung ultrasound examination. J. Matern. Fetal Neonatal Med..

[27] Liao D., Li J., Lv J., Sun T., Deng S. (2020). Evaluation of the Diagnostic Value of Peripheral Blood Parameters for Neonatal Pneumonia. Clin. Lab..

[28] Kumar C.S., Subramanian S., Murki S., Rao J.V., Bai M., Penagaram S., Singh H., Bondili P.B., Madireddy A., Lingaldinna S. (2021). Predictors of Mortality in Neonatal Pneumonia: An INCLEN Childhood Pneumonia Study. Indian Pediatr..

[29] Liu J., Lovrenski J., Hlaing A.Y., Kurepa D. (2019). Neonatal lung diseases: Lung ultrasound or chest x-ray. J. Matern. Fetal Neonatal Med..

[30] Zong H.F., Guo G., Liu J. (2019). Meta-analysis of lung ultrasound for the diagnosis of neonatal pneumonia. Chin. J. Pract. Pediatr..

[31] Leonart L., Tonin F., Ferreira V.L., Penteado S.T.D.S., Motta F.D.A., Pontarolo R. (2017). Fluconazole Doses Used for Prophylaxis of Invasive Fungal Infection in Neonatal Intensive Care Units: A Network Meta-Analysis. J. Pediatr..

[32] Antolín L.F., Sharland M., Warris A. (2019). Management of Invasive Fungal Disease in Neonates and Children. Pediatr. Infect. Dis. J..

[33] Liu J., Chen S.-W., Liu F., Li Q.-P., Kong X.-Y., Feng Z.-C. (2015). The Diagnosis of Neonatal Pulmonary Atelectasis Using Lung Ultrasonography. Chest.

[34] Liu J., Ren X.-L., Fu W., Liu Y., Xia R.-M. (2016). Bronchoalveolar lavage for the treatment of neonatal pulmonary atelectasis under lung ultrasound monitoring. J. Matern. Fetal Neonatal Med..

[35] Liu J., Zhao H.-R., Wei H.-L., Chen C., Qiu R.-X., Ren X.-L., Zhang L., Gao Y.-Q. (2020). Efficacy of Bronchoalveolar Lavage as Adjunct Therapy in the Treatment of Neonatal Severe Pneumonia: A Prospective Case–Control Study. J. Trop. Pediatr..

[36] Kurepa D., Boyar V., Zaghloul N., Beachy J., Zaytseva A., Teng D., Cooper R., Klewer S., Amodio J. (2021). Structured Neonatal Point-of-Care Ultrasound Training Program. Am. J. Perinatol..

